# Five different ways of reasoning: Tanzanian healthcare workers’ ideas about how to improve HIV prevention among same-sex attracted men

**DOI:** 10.1186/s12913-023-09771-3

**Published:** 2023-07-28

**Authors:** Alexander Mwijage Ishungisa, Elia John Mmbaga, Melkizedeck Thomas Leshabari, Britt Pinkowski Tersbøl, Kåre Moen

**Affiliations:** 1 Department of Behavioural Sciences, Muhimbili University of Health and Allied Sciences (MUHAS), Dar Es Salaam, Tanzania; 2 Department of Community Medicine and Global Health, Institute of Health and Society, University of Oslo, Oslo, Norway; 3 Department of Public Health, Global Health Section, University of Copenhagen, Copenhagen, Denmark

**Keywords:** Same-sex attracted men, Healthcare workers, Healthcare services, HIV, Access to care, Key Populations, Tanzania

## Abstract

**Background:**

Same-sex attracted men in Tanzania and globally carry a disproportionate burden of HIV. Drawing on qualitative research, this article explores healthcare providers’ ideas and recommendations regarding how to improve HIV prevention among same-sex attracted men.

**Methods:**

We carried out a qualitative study among healthcare workers in the cities of Dar es Salaam and Tanga in Tanzania between August 2018 and October 2019. Data were collected using qualitative methods of data collection, specifically in-depth interviews, focus group discussions, and participant observation. Study participants were recruited through a purposive sampling strategy that aimed to ensure variation in age, education, and work experience. Forty-eight interviews with 24 healthcare workers, six focus group discussions, and participant observation were conducted. A total of 64 persons participated in the study.

**Results:**

This paper describes five different “ways of reasoning” that were identified among healthcare workers regarding how to strengthen HIV prevention among same-sex attracted men. One held that punitive measures should be taken to prevent HIV transmission, another that health services needed to become more friendly towards men who have sex with men, a third that healthcare workers should reach out to provide more education to this population, a fourth called for strengthened collaboration between healthcare providers and same-sex attracted men in healthcare delivery, and the fifth proposed that activistic efforts be taken to remove structural barriers for same-sex attracted men to access healthcare.

**Conclusion:**

When reflecting on what is needed to strengthen HIV prevention among men who have sex with men, healthcare workers described six different ideas. One was that restrictive and punitive measures ought to be taken to prevent HIV transmission through same-sex sex. The remaining five promoted understanding of and support for same-sex attracted men. They prescribed more healthcare education, measures to improve attitudes among healthcare workers, healthcare delivery with user involvement, and political action to achieve law reform. Finally, some study participants raised concerns about the implementation of the national comprehensive package for key populations.

## Introduction

Drawing on qualitative research in Tanzania, this paper explores healthcare workers’ ideas and recommendations regarding how to improve HIV prevention among same-sex attracted men.

One of the reasons why we engage with this research theme is that the HIV situation among same-sex attracted men in Africa is still a considerable challenge. In Dar es Salaam, for example, the proportion of men who have sex with men and who are living with HIV (8.3% [1]) is twice the proportion in the general population (4.7% [2]). In some countries, moreover, a large and increasing proportion of new HIV infections occur among men who engage in sexual relations with other men. In Nigeria, for example, about 20% of all new HIV infections currently affect same-sex attracted men [3, 4].

The second reason why we are focusing on this topic is that previous research has demonstrated that there are many challenges relating to access to HIV services for same-sex attracted men in Africa. While healthcare workers on the continent have mixed attitudes towards same-sex attracted men [5], some men in this population encounter healthcare workers that are unsupportive [6–8]. Several studies have reported that there is stigma, discrimination, prejudice, mistreatment, harassment, abuse and rejection of same-sex attracted men in healthcare settings [9, 10]. Studies in Uganda and Ghana have found that some healthcare workers deny same-sex attracted men services altogether if they disclose that they are same-sex attracted men [11, 12]. Moreover, it has also been reported that same-sex attracted men are afraid they will be reported to the authorities by healthcare workers [13]. Consequently, many same-sex attracted men across Africa have limited access to HIV and sexual health services [14, 15]. People are worried about disclosing their information to healthcare providers [6, 16–20], and unsupportive attitudes among healthcare workers are reported to affect retention in and adherence to antiretroviral therapy [21]. Rather than seeking professional care, some men who engage in sexual relations with men resort to self-treatment [13, 22]. Instead of seeking professional healthcare, they diagnose themselves and/or buy drugs in medical stores and pharmacies [22, 23].

In some studies, healthcare workers’ lack of support has been linked to limited knowledge about men who have sex with men, their circumstances and healthcare needs [24] and deficient or inappropriate training [25–30]. Several other challenges have also been reported, varying from forms used in the clinic that lack questions about issues that are important to ask same-sex attracted men [31] to a lack of funding [32, 33]

We are not aware of previous papers that have devoted themselves to exploring the different types of recommendations healthcare workers may have regarding HIV prevention among same-sex attracted men. However, a review of the literature found several publications that included recommendations from healthcare workers about things to consider in efforts to improve HIV prevention and reduce the HIV burden in this group. Some have emphasized the need for improved relationships between healthcare providers and men who have sex with men [26, 34] and changing healthcare providers’ attitudes towards same-sex attracted men through targeted training [21].

## Methods

The present article draws on qualitative research with healthcare providers working in the cities of Dar es Salaam and Tanga in Tanzania. The two cities were selected because previous work had demonstrated that same-sex attracted men there carry a substantial burden of HIV [35, 36], and in part because there are considerable population of such men in these locations [37, 38]. This study employed an ethnographic design and fieldwork involved in-depth interviews, focus group discussions and participant observation (see Table 1). The data collection methods complemented each other, as will be evident in the results section. Data collection was carried out between August 2018 and October 2019.

**Table 1:** Distribution of study participants, by city and the research activities they were part of

	Participated in IDI only	Participated in FGD only	Participated in PO only	Participated in IDI + FGD	Participated in both IDI + PO	Participated in both FGD and PO	Participated in all three methods	Total participants in each region
Dar es Salaam	5	14	5	8	2	2	0	36
Tanga	3	14	3	5	1	2	0	28
**Total**	**8**	**28**	**8**	**13**	**3**	**4**	**0**	**64**

The first author, during the time of data collection, interacted with and conducted interviews and group discussions with healthcare providers of different cadres (see Table 2) who worked in health facilities or in non-governmental organizations implementing HIV related projects.

**Table 2:** Distribution of study participants according to their cadres

Cadre	No of participants	Facility of work		Trained on KVP	
		**Public**	**Private**	**yes**	**no**
Medical doctors (MD)	10	6	4	3	7
Assistant MD	16	5	11	3	13
Clinical Officers	24	11	13	8	16
Enrolled Nurses^a^	14	6	8	3	11
Total	64	28	36	17	47

^a^The difference between assistant medical doctors (MDs), Clinical officers (CO), Registered nurses (RN), and enrolled nurses (EN) are in qualification and preparation. Enrolled nurses complete a diploma of nursing which is a two years course, and Registered nurses complete a bachelor’s degree in nursing which is a three years course. Clinical officers. Clinical officers are non-physician clinicians. Experienced clinical officers may enroll in an advanced diploma which takes two years to complete, and become assistant medical officers

In total, 64 healthcare workers participated in interviews, focus group discussions and/or participant observation as indicated in Table 1 below. The study participants were of different age, and all had at least one year of work experience. Some had good and long experience with taking care of patients belonging to same-sex attracted men’s population, others had little or none. Seventeen had received training on how to provide healthcare to key and vulnerable populations, the others had not. We used a purposive sampling strategy to recruit participants with the aim to maximize variation in experiences and perspectives. Differences in age, education, work experience, level and location of healthcare facilities, and the type of services offered were taken into consideration when selecting study participants.

### Qualitative interviews

A total of 24 persons took part in in-depth interviews. Each person was interviewed twice so that a total of 48 interviews were conducted. 13 of the interviewees also participated in a focus groups discussion and three engaged with the researcher during participant observation. Swahili was the language used, and most interviews lasted between one and one and a half hours. All interviews were audio-recorded and transcribed verbatim and later translated from Swahili to English.

### Participant observation

The first author took part in daily activities and events together with healthcare providers and same-sex attracted men seeking healthcare services. He joined some healthcare workers at their places of work and engaged in discussions about topics related to healthcare for men who have sex with men. He also took part in outreach services targeting same-sex attracted men during the day and night in the two cities on six occasions. Some healthcare workers who interacted with the first author during fieldwork took part in the outreach activities. During his participation in these events, he took scratch notes which were later expanded into fieldnotes. Also, some discussions during outreach were audio recorded and later transcribed. In total, fifteen healthcare workers were encountered during participant observation. Of these, three also participated in interviews for this study, and four in focus group discussion.

### Focus group discussions

We conducted six *focus group discussions* (three in each city) to further explore some of the themes and topics that came up during individual interviews and participant observation. In total, 45 healthcare workers participated in focus group discussions and among these, thirteen had participated in interviews while four had participated in participant observation (see Table 2). One group discussion comprised of healthcare providers working in private health facilities, one of providers working in publicly owned facilities, two of personnel who had been trained on serving same-sex attracted men, and two of providers who had not received this kind of training. The number of participants in each group discussion ranged from seven to eight. Each discussion lasted between one and two hours. All discussions were semi-structured and guided by a set of questions prepared prior to the event. Questions asked Included: what should be done to increase acceptability of same-sex attracted men in healthcare, what should be done to improve HIV prevention for same-sex attracted men, and what should be done to improve healthcare workers’ capacity to provide healthcare to same-sex attracted men. Swahili was the language used and all sessions were audio-recorded and transcribed verbatim.

Data analysis: We carried out content analysis [39] of interview transcripts and fieldnotes. Open coding, an analytical process where concepts (codes) are identified from the qualitative data, described and named [39] was applied in the initial stage to identify emerging themes. Example of codes that emerged during the analysis include ‘make MSM wait longer than others’, ‘force MSM live in a special area’, and ‘educating same-sex attracted men’, ‘improving attitudes of healthcare providers’ and ‘improving health services’. Those codes that were related, were later subsumed into broader thematic categories. For this paper, we draw on portions of the material that fell into a category we referred to as ‘healthcare workers' recommendations on how to improve HIV prevention among same-sex attracted men’.

We obtained ethical clearance from the MUHAS Institutional Ethics Review Board (DA.251/267/01.C110), and then community entry permits from the Ministry of Regional Administration and Local Government as well as from Dar es Salaam and Tanga regional authorities. All participants provided written informed consent prior to participation in the study. All study participants approached consented to participate. No direct personal identifiable information was recorded except study participants’ voices. Recorded conversations were later transcribed and later data and recordings were stored in a secure offline computer. To ensure participants’ anonymity, this paper uses pseudo names to refer to participants who took part in this study.

## Results

The following describes the various recommendations provided by healthcare workers for measures they thought should be taken to improve HIV prevention among same-sex attracted men.

### Strengthening HIV prevention through punitive style measures

Some healthcare workers recommended the use of force and punishment in order to help prevent HIV. One of the mentioned recommendations in this category was to isolate men who were HIV positive. One of the reasons to isolate MSM was that they were perceived to have a tendency of changing partners often and hence it could be easy to transmit HIV to the general population if some they are infected.

*“In my view, the government needs to make a difficult decision and allocate a special place where HIV infected mashoga*^1^*can settle. That will limit their interaction with other, non-infected members of society, and the virus will not cross to the general population and HIV negative mashoga” Hadija, in interview (IV)12*

Another idea mentioned was to deny access to healthcare services for same-sex attracted men. The rationale proposed was that this would scare men away from engaging in same-sex practices that might put them at increased risk of HIV infection.

*“When msm know that there are services for them and that providers are there to help them, they will not stop putting themselves at risk of HIV. But if we deny them health services, they will fear the infection and stop same-sex practices. If their sexual practices are allowed to continue unrestrained, they will cost resources, energy and time from the government and providers” Halima, IV7*

### Strengthening HIV prevention through education

While there were thus some recommendations that promoted punishment of men sexing with other men, many more focused on providing men with better information, education, and communication. Many of the recommended approaches were of the type that would aim to help men change behaviours.

Among them was that same-sex attracted men should be encouraged to reduce the number of sexual partners,

*“Most mashoga lack sexual education and change their sexual partners often. That is the reason why they are contracting HIV and STI so easily. To prevent such infections, having education and being faithful to their partners is the best solution” Huruma, IV13*

Taking it a step further, some study participants argued that men should be advised to stop same-sex sexual practices altogether,

*“Nothing else than stopping homosexual sex can reduce the risk of HIV infection among mashoga. Although there may be risks of infection in heterosexual sex, somehow the risk is lower than in same-sex practices” Alan, in group discussion (GD) 4*

In opposition to views like this, other study participants argued that sexual health education should focus on promoting protected sex,

*“Look here [...], in the current world, sex is a source of income. We will be cheating ourselves if we stress on faithfulness, it will not work. What is important is to give education and stress the use of protected sex. With that, many mashoga*^*1*^* will remain free from HIV and STI” Baraka, IV19*

Another topic that was recommended for inclusion in health education targeting men who have sex with other men was the benefits of early healthcare seeking,

*“Msm are often late to come for care when they have HIV or STIs. By the time they come, they are at the late stages of the disease, and they may have spread it to many others. To stop such infections, MSM should be educated to report to healthcare facilities as early as possible when they suspect infections” Marando, GD6*

A final topic that was recommended was that MSM should be encouraged to engage in open and transparent communication with healthcare workers. It was pointed out that in fear of stigma and discrimination in healthcare settings, many men engaging in same-sex sex conceal or distort information about their sexual identity, their sexual practices and their health problems, and that they consequently may receive ill-suited advice or incorrect treatment.

*“The main problem facing mashoga who seek care is that they hide their needs and health problems. They don’t open up to providers about what troubles them. I think more education is needed to help them communicate their exact health needs and problems when they meet providers, otherwise providers will not know.” Samson, IV14*

### Strengthening HIV prevention through improved knowledge and attitudes among healthcare workers

A third category of recommendations were those that focused on healthcare workers themselves and how they as a group should improve their approach to men who are sexually attracted to other men. To contribute to prevent HIV among men this group, healthcare services had to be experienced as friendly. Nathan was an example of providers who had such perspectives.

*[..] to reduce the risks of HIV among MSM, providers must change their attitude towards them. If they are friendly to MSM, they will give them good care, there will be no stigma, and many mashoga will come to seek care. I think everyone likes going to facilities with providers who are friendly and caring.” Nathan, IV20*

Some participants complained that healthcare providers were insufficiently prepared to handle the healthcare needs among same-sex practicing men and recommended that training be provided.

*“A main problem is that many of us lack training on HIV and the circumstances that mashoga pass through. That is why there is high stigma in facilities and among providers. To be able to end HIV among mashoga, providers need to be trained not only on HIV, but also on their needs and on different techniques of engaging mashoga in care delivery” Mtitu, GD2*

Some participants said that many healthcare workers lack knowledge about who same-sex practicing men are, their healthcare needs, and their position in the HIV epidemic. To improve HIV prevention, there was a need to increase their understanding of this group of men so that they would treat them more like any other member of society. Francis was an example of a study participant who held this view:

*“Most healthcare providers do not understand who msm are and their position in the HIV epidemic. To end HIV, providers must understand that msm are people like other members of society, deserving care and life. And that they have positive contributions in the welfare of this nation. Their health needs and problems must be addressed, like those of other members” Francis, IV16*

Some healthcare workers recommended that anti-stigma campaigns and education activities be carried out in the clinics since stigmatizing attitudes among healthcare workers (and among other patients) worked to block same-sex practicing men from care.

*“It is important for every facility to have anti-stigma campaigns to change attitudes of providers and patients about mashoga and at the same time attract mashoga to get care. Because stigma is a big reason for mashoga not to come for care and hence to remain at risk of infection.” Wambura, GD4*

### Strengthening HIV prevention through user involvement

Some providers argued that healthcare workers should work together with same-sex attracted men in the development and delivery of care. Two rationales were given for this argument. One was to increase the sense of ownership of services by men who have sex with men and a second was workers might change their attitudes through interaction with men who have sex with men.

*“To eliminate HIV among mashoga, it is important that mashoga themselves be involved in healthcare development and delivery. When they are involved, they trust and own that care, and through such interaction, other health workers will change their perceptions and attitudes towards mashoga. And most of all, they can also advocate for more of their care demands to be available in health facilities than now” Matogo, IV15*

### Strengthening HIV prevention through societal measures

As described just above, some healthcare workers recommended that measures to reduce stigma and discrimination be implemented in healthcare facilities. Others argued that one needed to take a broader approach and work towards reduced stigma and discrimination on a broader societal level. The rationale of this argument was that reducing stigma and discrimination would increase access to both preventive and curative services.

*“If we want to prevent the risk of HIV and other infections in these populations, much work is needed to reduce or eliminate stigma and discrimination, not only among healthcare workers but also among people surrounding them. With that, mashoga will have a chance to express their needs and problems and access right services” Bruno, IV22*

Several participants also argued that there was a need to overhaul national laws and policies. In Tanzania, the penal code [40] prescribes that “carnal knowledge against the order of nature” is a crime punishable by thirty years to life imprisonment. Several study participants pointed out that this law creates fear that prohibits men who have sex with other men from seeking care and disclosing their health needs.

*“[..]much effort is needed to change the laws prohibiting sexual relationships between men because it is fear of the laws that make mashoga not seek healthcare or disclose their healthcare needs and problems.” Adoroph, IV18*

Adoroph continued by adding that changing the law should go hand in hand with changes in health policies to better accommodate the health needs of men engaging in same-sex sex.

*But also, health policies need to be changed to accommodate the health needs of mashoga compared to how they are now. If laws and policies are changed, good interaction between mashoga and health workers will be enhanced, providers and the whole community will change the attitudes towards mashoga. It will increase access to care in this population” Adoroph, IV18*

### Strengthening HIV prevention through improved health services

Study participants also pointed out that in order to improve HIV prevention among same-sex attracted men, relevant services must be available in the first place. They highlighted that important drugs and laboratory services needed by same-sex practicing men were either missing or hard to access or expensive.

*“In my opinion, if we want to control HIV, we need to provide all sexual health services free of cost. These are services that are not provided in many facilities, one must have financial muscles to get them. But as you know, most mashoga are very poor, and many cannot even afford their meals. Will they pay for such services? If these services were free and available in many facilities, then we could be sure that mashoga get them and protect themselves not only from HIV but also from other infections” Francis, IV16*

Several study participants asked critical questions about the so-called comprehensive HIV package for same-sex attracted men (CHIP). The CHIP is a package of healthcare services that men who have sex and other key populations are supposed to receive [41], but several study participants questioned its availability.

*“I have never seen and used the CHIP, and I know most of us [providers] have not seen or used it, and don’t know what is contained in it.” Kamanzi, PO 2*

*“As a caregiver, I understand how much at risk mashoga are when we think of the HIV epidemic, and this is why the special package of care was established. But you will be surprised that most health facilities do not offer the services outlined in the package.” Mangura, IV21*

Some healthcare workers argued that pre-exposure prophylaxis (PrEP) should be available in most healthcare facilities.

*“Important drugs that would protect mashoga from HIV are not offered in many health facilities including my own. For example, PrEP is not provided in many facilities. And I can assure you in those facilities where they have been supplied, they are not there all the time when needed.” Winfrida, IV24*

Some study participants pointed out that most healthcare workers do not understand the importance of PrEP. They pointed out that this lack of understanding leads to under-prescribing and a slow roll-out of PrEP to men who have sex with men.

*“I can assure you on this, most of healthcare providers do not understand what PrEP is, and its importance for mashoga and in controlling HIV epidemic. We have never been trained on issues of PrEP, that is why such drugs are not given to mashoga and providers can’t push the government and management in their facilities [health] to ensure the drugs are available all the time” Dorosera, IV4*

## Discussion

The healthcare workers who participated in this study provided a range of perspectives and recommendations on how to improve HIV prevention among same-sex attracted men. Below, we summarize their different views, perspectives and recommendations as five distinct models or ideas – five different ways of reasoning – that existed among healthcare providers in Tanzania.

The first may be referred to as a “punitive model” of HIV prevention because it emphasized that there ought to be punishment and restrictions for men who engage in same-sex practices. Interestingly, healthcare providers had two rather different rationales for this proposal. Some considered same-sex sexual practices to be morally wrong, whereas others primarily reasoned that avoidance of same-sex practices would protect men from contracting HIV. To punish same-sex practices between men were thus variously thought of as a strategy to discourage homosexual behaviour and as a strategy to stop the spread of HIV. Among the punitive approaches recommended were to isolate same-sex practicing men who are HIV positive and to limit healthcare services to men who have sex with men. It was argued that actions like these would lead to less HIV transmission, and that they would reduce both the burden of HIV among men who have sex with other men and the resources spent on HIV.

We refer to the second way of reasoning as the “friendly services model” because it emphasized that healthcare workers should receive men who have sex with other men with kindness and friendliness. The key message in this model stood in clear opposition to the punitive model as it emphasized that healthcare workers should accept and have positive attitudes towards same-sex practicing men. Study participants emphasized that healthcare providers need to allow men who have sex with men to freely and unconditionally contact them for consultation whenever they have health problems. To be able to develop more friendly attitudes, some study participants said that healthcare providers need to receive training on how to recognize, relate to, engage with and take care of same-sex loving men.

The third way of reasoning we identified could be referred to as an “educational outreach model” because it emphasized that healthcare workers should reach out to same-sex attracted men to provide education on different relevant topics. In this model, knowledge building was considered vital for the health of men who have sex with men. Among topics that were thought of as salient to focus on in education were the advantages of partner reduction and protected sex as well as early health seeking behaviour.

We refer to the fourth way of reasoning as the “collaboration model” because it highlighted that same-sex attracted men should be invited to take part in the development and delivery of healthcare services. The collaboration model goes beyond the friendly and outreach models in the sense that it does not only promote that men who have sex with men should be welcomed to healthcare (as in the friendly services model) or given targeted education (as in the educational outreach model), but that they should also be given power to take part in the shaping of the health services for their group. As opposed to the previously mentioned models, same-sex practicing men are no longer considered passive recipients of care developed and delivered by others; they are becoming active participants in the production of care. An additional rationale for the collaborative model was that it would lead to improved understanding of men engaging in same-sex sex among healthcare workers and thereby strengthen their competence and capacity as healthcare providers for this group. In other words, collaboration was in a sense regarded as a way of empowering both patients and healthcare workers. Finally, the collaboration model was also thought to make same-sex attracted men feel a sense of belonging to and ownership of healthcare and to have the potential to increase the trust between patients and providers.

The fifth and last idea we identified could be referred to as an “activistic model” because it promoted actions on a system and/or political level that aim to enhance the understanding, acceptance and inclusion of same-sex attracted men in society (including in the healthcare services). Some study participants pointed out that important changes needed to be taken at structural level to create better conditions for men who have sex with men, including changes in laws and policies that negatively affect men who engage in same-sex practises. The activistic model also extended some of the arguments in the previously mentioned models. For example, some of the study participants insisted that not only should friendly sexual healthcare services be available to men loving other men, they should also be offered free of charge (since cost was thought to be a barrier against access to care).

This diversity of perspectives and opinions among the healthcare workers who took part in this study stands in contrast to much reporting that risks giving the impression that practically all healthcare workers in Africa are utterly homophobic [42, 43]. This is clearly not in line with what we found. While on the one extreme of the spectrum we did find healthcare workers who held very disapproving views indeed, all the other four models we describe express varying degrees of understanding of and support for same-sex attracted men.

While punishment and restriction was thought by some study participants to be a strategy that could lessen the impact of HIV on men who have sex with men, Fagan and Meares are among those who argue why and how such approaches may be counter-productive [44, 45]. Instead of achieving any intended benefit, punishment is always accompanied with risks, pertaining not only to the individuals who experience them, but also to their neighbouring members of society [45]. For example, punishing men who have sex with men with the intention of changing them undoubtedly risks pushing them away from healthcare and from openly mentioning their sexual orientation to healthcare providers. In such a situation, much of what has been achieved through HIV programming could be lost and the consequences might affect entire communities with the result that the HIV pandemic could begin to rise after years of declining. An even more fundamental issue is the obvious conflict between punitive approaches and the clinical-ethical principle of respect for the patient’s autonomy, which promotes the idea that everyone “should have the power to make rational decisions and moral choices, and each should be allowed to exercise his or her capacity for self-determination”[46].

However, while some healthcare providers advocated for punishment; many did not. As we will show elsewhere, a recent study among healthcare providers in Dar es Salaam and Tanga, Tanzania (Ishungisa et al., forthcoming) indicate that a majority are supportive of same-sex attracted men who seek healthcare services. There have also been studies elsewhere in Sub-Saharan Africa that have found supportive attitudes to be common among healthcare providers towards same-sex practicing men [25, 26, 47, 48]. Friendly engagement with, acceptance of, and collaboration with this population not only has the potential to contribute to increased trust in healthcare services, it may also empower patients and give them a feeling of ownership of care [49–51]. As pointed out by the World Health Organization (WHO 2017) and Delaney [52], potential advantages of accepting attitudes and collaboration approaches are that they may enhance the safety of patients and reduce unnecessary harm [52]. Other advantages of accepting and collaborating with patients in delivering care is that collaboration acknowledges patients’ preferences and values, promotes flexibility of care, increases patients’ satisfaction, self-efficacy and autonomy as well as leads to improved health outcomes [52–55]. In his “Principles of clinical ethics”, Basil Vakey [46] pointed out that healthcare providers have a professional obligation to work to the benefit of patients as well as avoid harming them [46].

Those subscribing to what we call the activistic model had in mind that laws and policies that prohibit same-sex sexual practices need to be reversed to provide space for provision of healthcare service. This aligns with the guidelines of the World Health Organization (WHO) which stipulate that in countries where anti-homosexuality laws and policies are used to deter access to healthcare and health information, such laws need to be reversed [56], and in circumstances where they are not, healthcare professionals need to follow their obligations and ethics of saving patients’ lives [56].

Lastly, we note the study participants’ views on the so-called comprehensive package of HIV intervention package for key populations (CHIP), which was launched in Tanzania in 2014 [57] and revised in 2017 [41]. Several study participants pointed out that the CHIP remains to be fully implemented. They were of the impression that many of the services outlined in the package are not provided, and the ones offered are not consistently available when needed by men sexing with other men. These reports may seem to indicate that there could be a problem with the implementation of the health authorities’ own strategy for healthcare for key populations in Tanzania. There would therefore seem to be a need for an urgent evaluation of the performance of the CHIP.

## Conclusion

This paper has explored Tanzanian healthcare providers’ views on measures they thought should be taken to strengthen HIV prevention among men who have sex with men. This is of interest because men who engage in sex with other men are still experiencing a considerable HIV epidemic in Tanzania, and because previous research has demonstrated that there are many challenges relating to access to HIV-related health services. As we have seen, healthcare workers had diverse ideas, and we categorized them as six different ‘models’ – or ‘ways of reasoning’. One way of reasoning was that restrictive and punitive measures ought to be taken to prevent HIV transmission through same-sex sex. These are clearly not in line with either national policies or best practices promoted by global agencies such as WHO and UNAIDS. The remaining five models we identified promoted understanding of and support for same-sex attracted men. They prescribed more healthcare education, measures to improve attitudes among healthcare workers, healthcare delivery with user involvement, and political action to achieve law reform. Finally, some study participants raised concern about the implementation of the national comprehensive package for key populations. They had not heard of the package and suggested that it might exist mostly on paper. To address HIV epidemic among same-sex attracted men’s population, positive and inclusive healthcare workers’ perspectives and recommendations on how HIV prevention need to be integrated in the planning, organization and delivery of HIV care.

## Data Availability

The dataset generated during and/or analyzed during the current study are not publicly available due to the sensitivity of the topic as part of our strategy to ensure the anonymity of our participants. In the case of questions, please contact Alexander Mwijage Ishungisa (mwijagealexander2014@yahoo.com).

## Footnotes

1 *Mashoga* is a Swahili word (plural) used to refer to men who engage in same-sex sexual practices. *Mashoga* is normally meant to include men who take the receptive position in sex with men.
